# Metabolic-stem cell crosstalk in PD: NK1 cells as key mediators from a bioinformatics perspective

**DOI:** 10.3389/fneur.2025.1681261

**Published:** 2025-11-13

**Authors:** Junxin Zhao, Yibiao Chen, Lundeng Hu, Shuna Huang, Qibin Zheng

**Affiliations:** 1 First Affiliated Hospital of Fujian Medical University, Fuzhou, China; 2 Department of Neurosurgery, Binhai Branch of National Regional Medical Center, The First Affiliated Hospital, Fujian Medical University, Fuzhou, Fujian, China

**Keywords:** stem cell, metabolic, Parkinson’s disease, NK cell, CA4, inflammation

## Abstract

**Introduction:**

Parkinson’s disease (PD) is characterized by progressive degeneration of dopaminergic neurons in the substantia nigra and pathological aggregation of α-synuclein. Although existing therapies alleviate clinical symptoms, however, due to the unclear etiology, it remains impossible to completely halt this process through currently available approaches. This study aims to elucidate molecular mechanisms underlying PD pathogenesis and identify novel candidate biomarkers.

**Methods:**

We integrated bioinformatics analysis of GEO datasets to pinpoint pivotal genes in PD progression from metabolic and stem cell perspectives. Hub genes were empirically validated using quantitative real-time polymerase chain reaction (qRT-PCR) and western blotting in animal specimens. A combinatorial predictive model was constructed and evaluated via nomogram. Single-cell RNA sequencing (scRNA-seq) data from PD cohorts were interrogated to localize cell-type-specific expression patterns of signature genes and delineate subtype-specific mechanisms. Our analytical workflow entailed: differential expression screening, functional enrichment, protein–protein interaction (PPI) network construction, and machine learning (ML) algorithms.

**Results:**

Our study reveals BMX and CA4 as key hub genes. Experimental confirmation of their dysregulation in in vivo PD models. Development of a high-accuracy PD prediction model (AUC >0.6). scRNA-seq analysis identified an NK cell subtype (NK1) enriched with CA4 expression. KEGG pathway analysis of NK1 marker genes implicated their role in neuroimmune crosstalk during PD progression.

**Discussion:**

This work establishes a novel CA4-NK1-PD axis, providing a potential therapeutic entry point for future interventions.

## 1 Introduction

PD is a neurodegenerative disorder marked by the progressive loss of dopaminergic neurons in the substantia nigra and the abnormal aggregation of α-synuclein (α-syn). Despite recent advances in PD research, due to the unclear etiology (1), it remains impossible to completely halt this process through currently available approaches. Standard treatments, including levodopa, dopamine receptor agonists, deep brain stimulation (DBS) of the subthalamic nucleus (STN) or internal globus pallidus (GPi), and rehabilitative interventions, frequently lead to motor complications (such as motor fluctuations and dyskinesia), neuropsychiatric side effects (such as schizophrenia), and limited effectiveness for non-motor symptoms (such as sleep disorders, olfactory dysfunction, autonomic dysfunction, and cognitive and neuropsychiatric disturbances) (2). Therefore, it is crucial to clarify the molecular mechanisms of PD pathogenesis and discover new biomarkers, which are vital for early diagnosis, targeted treatment, and prognostic assessment.

Metabolism is fundamental to cellular function and homeostasis, especially in the energy-intensive central nervous system (CNS). CNS energy metabolism is heavily reliant on mitochondrial oxidative phosphorylation. In Parkinson’s disease, dopaminergic neurons in the substantia nigra show significantly decreased mitochondrial complex I activity, resulting in impaired ATP production and increased reactive oxygen species (ROS) generation, which perpetuates a cycle of metabolic dysfunction and oxidative stress (3). Additionally, dysregulated lipid metabolism—for example, ceramide accumulation—has been shown to facilitate α-synuclein oligomerization (4), while iron dysregulation exacerbates neuronal loss through ferroptosis (5, 6).

Stem cell-based therapies have recently emerged as a promising and potentially transformative approach for treating Parkinson’s disease. By differentiating into functional dopaminergic neurons, secreting neurotrophic factors, and modulating neuroinflammation, stem cells offer novel avenues to reconstruct the damaged basal ganglia circuitry (7, 8). These strategies address two fundamental limitations of traditional therapies: (1) the irreversible loss of dopaminergic neurons, necessitating cellular replacement to restore striatal dopamine levels; and (2) the need for neuroprotection and microenvironmental repair, including suppression of chronic inflammation and oxidative stress, to slow disease progression. However, it remains unclear whether neural stem cells are involved in PD pathogenesis (9) or whether metabolic alterations and stem cell dynamics interact during the disease course (10).

This study explores PD through metabolic and stem cell lenses by integrating bioinformatics analyses of single-cell and bulk RNA sequencing data. We identified key genes and cell types associated with PD progression and validated the diagnostic performance of a gene-based classifier using SHAP modeling and nomogram construction. We investigated the involvement of natural killer (NK) cells in the pathophysiology of PD and analyzed the importance of increased CA4 expression in these cells to understand the molecular immune mechanisms in PD. Collectively, our findings offer novel insights that may inform future research and therapeutic strategies for Parkinson’s disease.

## 2 Methods

### 2.1 Data acquisition and preprocessing

The Gene Expression Omnibus (GEO) database provides a comprehensive repository for microarray and high-throughput sequencing data (11). Raw expression data from four GEO datasets—GSE99039, GSE18838, GSE6613, and GSE57475—were retrieved. The raw CEL files were processed using the “affy” R package (v1.74.0, https://bioconductor.org/packages/affy) (12), which includes background correction, normalization, and probe summarization. Corresponding platform annotation files (GPL570, GPL5175, GPL96, and GPL6947) were downloaded to map probe IDs to gene symbols. Probes without corresponding gene symbols were excluded, and for genes with multiple probes, the average expression value was used to represent the gene.

The training dataset was created by merging GSE99039 and GSE18838, followed by batch effect removal using the “sva” package (v3.50.0) (13). GSE6613 and GSE57475 served as external validation datasets. The “limma” package (v3.52.4, https://bioconductor.org/packages/limma) (14) was utilized to identify differentially expressed genes (DEGs) between PD and control samples. Genes with *p* < 0.05 were considered differentially expressed (15).

### 2.2 Pathway enrichment analysis

Gene set enrichment analysis (GSEA) (16) assessed the statistical significance of differences in predefined gene sets between PD and control conditions. The “h.all.v7.5.1.symbols” hallmark gene set was downloaded from the Molecular Signatures Database (MSigDB) (17), and subjected to GSEA analysis with significance defined as *p* < 0.05. The “clusterProfiler” package (v4.10.0, https://bioconductor.org/packages/clusterProfiler) (18) was utilized for functional enrichment analyses, such as Gene Ontology (GO) and Kyoto Encyclopedia of Genes and Genomes (KEGG) pathway analysis, to investigate the biological processes linked to potential therapeutic target genes. GO terms were classified into biological process (BP), cellular component (CC), and molecular function (MF), with significance determined by an adjusted *p*-value of less than 0.05.

### 2.3 WGCNA identifies pathogenic genes

Based on transcriptomic data from the training set, with PD and Control groups assigned as phenotypic traits for WGCNA, the expression matrix of all genes was used as input. Weighted Gene Co-expression Network Analysis (WGCNA) is a method used to identify clusters (modules) of highly correlated genes, summarize these clusters using the module eigengene or an intramodular hub gene, relate modules to one another and to external sample traits, and calculate module membership measures. This approach helps in understanding the correlation patterns among genes across microarray samples.

The “WGCNA” package (v1.71) (19) was utilized to conduct WGCNA on the training dataset, aiming to identify co-expression gene modules linked to PD. All expressed genes were used as input, with phenotype traits defined by PD versus control status. Sample clustering was applied to detect and remove outliers. The soft-thresholding power *β* was chosen to ensure scale-free topology. Modules were constructed with a minimum module size of 200 genes. The modules most positively and negatively correlated with PD were retained, and their constituent genes were used for downstream analysis.

### 2.4 Identification of PD-associated stem cell and metabolic genes

Metabolism-related genes were collected from MSigDB by querying for the keyword “metabolism” across HALLMARK, KEGG, and REACTOME gene sets (17). Stem cell-related genes were obtained from the StemChecker database, which includes 26 curated gene sets (21). Intersections were computed between DEGs, PD-associated module genes (from WGCNA), metabolic genes, and stem cell genes. The overlapping genes were analyzed for GO and KEGG enrichment using the “clusterProfiler” tool (18).

### 2.5 Construction of protein–protein interaction network

To investigate the protein interactions among intersecting genes, the STRING database (v12.0) (20) was used to construct a PPI network using a combined score >0.15 as the threshold. Key clusters were identified using the MCODE plugin (v2.0.3) in Cytoscape v3.10.2 (22). Four topological analysis algorithms—MCC, MNC, Degree, and EPC—implemented in the cytoHubba plugin (v0.1) (23) were employed to rank the top 30 hub genes. Genes common to all four rankings were defined as final hub genes.

### 2.6 Feature gene selection and diagnostic model construction via machine learning

Twelve machine learning algorithms were employed to identify robust diagnostic gene signatures: Random Forest (RF), Least Absolute Shrinkage and Selection Operator (Lasso), Ridge, Elastic Net (Enet), Stepwise GLM, Support Vector Machine (SVM), glmBoost, Linear Discriminant Analysis (LDA), Gradient Boosting Machine (GBM), eXtreme Gradient Boosting (XGBoost), and Naive Bayes. The models underwent 10-fold cross-validation training on the training dataset and were assessed using validation datasets. The model’s diagnostic accuracy was evaluated using the area under the receiver operating characteristic curve (AUC), with the model exhibiting the highest mean AUC across validation datasets chosen for further analysis.

### 2.7 Validation of feature gene expression and ROC analysis

The expression of feature genes was compared between PD and control samples using the Wilcoxon test. The “pROC” package (v1.18.5) (24) was utilized to compute ROC curves and AUC values for assessing diagnostic performance in both training and validation datasets. Genes with *p* < 0.05 and AUC >0.6, and consistent expression trends across datasets, were retained for downstream analysis.

### 2.8 Immune cell infiltration and correlation with feature genes

Given the role of immune infiltration in PD pathology, immune cell fractions were estimated using the CIBERSORT algorithm (25). Differences in immune cell composition between PD and control groups were analyzed, and Spearman correlation was used to assess associations between immune cells and diagnostic genes. Heatmaps were generated to visualize correlation patterns.

### 2.9 Molecular mechanisms underlying diagnostic scores

GeneMANIA (26) was utilized to construct co-expression networks for investigating potential biological interactions of diagnostic markers. PD samples were divided into high- and low-score groups using the median diagnostic score as a threshold. GSEA was then performed to identify enriched pathways. Additionally, hallmark pathway enrichment scores were calculated using the GSVA algorithm (27), and differential enrichment was tested using “limma.” Correlations between diagnostic scores and hallmark gene sets were also assessed.

### 2.10 SHAP-based model interpretation

To interpret the final prediction model, we applied the SHAP (SHapley Additive exPlanations) algorithm. Global interpretations were visualized using SHAP summary plots, which illustrate the mean contribution of each feature to the model, thereby characterizing the model’s overall behavior. SHAP was applied to the baseline model to address both regression and classification tasks (28).

### 2.11 Nomogram construction

A diagnostic nomogram for PD was developed using the characteristic genes and their expression levels from both control and PD groups. The nomogram was developed using the “rms” package (Version 6.8-1) in R (29). The nomogram represents a regression model by assigning scores to predictors according to their regression coefficients. A total score is then calculated for each subject and translated into a predicted probability of PD occurrence through a mapping function. Calibration and decision curve analyses evaluated the model’s accuracy and clinical utility.

### 2.12 Single-gene GSEA analysis

Samples in the training cohort were categorized into high- and low-expression groups according to the expression levels of the selected signature genes. GSEA utilized the “limma” algorithm to calculate log fold changes between the groups. The reference gene set used was “c2.cp.kegg_legacy.v2023.2.Hs.symbols.gmt,” with a significance threshold of *p* < 0.05.

### 2.13 Forecasting drug interactions and molecular docking

Candidate drugs targeting the identified signature genes were sourced from the DrugBank database^1^ (30). Molecular docking analyses were performed using AutoDock. Protein crystal structures were obtained from the Protein Data Bank (PDB, https://www.rcsb.org) (31). PyMOL was used to remove water molecules and native ligands. Proteins were prepared using AutoDock Tools by adding hydrogens, calculating charges, and merging nonpolar hydrogens. Docking simulations were executed in AutoDock Vina by setting appropriate grid box sizes and genetic algorithm parameters. Visualization of docking results was conducted in Discovery Studio 2019 (32). Drug structures were sourced from the PubChem database (33).

### 2.14 Single-cell RNA-seq data analysis

The GSE157783 PD single-cell transcriptomic dataset was analyzed with the Seurat package (Version 4.3.0, https://cran.r-project.org/web/packages/Seurat/index.html) (34). Quality control was performed with thresholds of nFeature_RNA >200 and <7,000, and cells with >20% mitochondrial gene expression were excluded. PCA was performed using the 2,000 most variable genes. The Harmony algorithm was employed to correct batch effects. Dimensionality reduction was performed using UMAP on the top 20 principal components, and clustering was subsequently conducted at a resolution of 0.5.

Cell clusters were annotated using literature and marker genes from the CellMarker 2.0 database (35), a detailed resource of experimentally validated markers for human and mouse tissues. Diagnostic scores for individual cells were assessed using GSVA, based on the expression levels of identified signature genes. Cells with the highest diagnostic scores were selected for subsequent analyses.

### 2.15 Cell–cell communication and ligand–receptor interaction analysis

Intercellular communication between cell populations was inferred using the CellChat R package (Version 1.6.1) (36). This framework forecasts interaction strength by analyzing the expression levels of immune-related ligands and receptors. CellChat contains a curated database encompassing multimeric ligand–receptor complexes, soluble agonists/antagonists, and membrane-bound co-receptors with activating or inhibitory functions. Interaction inference involved the identification of differentially expressed signaling genes, integration of average expression and communication probabilities, and determination of statistically significant communication events. Communication networks were compared between normal and PD samples across cell types.

### 2.16 Subpopulation analysis of high-scoring cells

Subcluster analysis was performed on cells with the highest diagnostic scores. Cell identities and subclusters were annotated using marker genes from CellMarker 2.0 and relevant literature (37, 38). Subclusters were defined based on differentially expressed top marker genes. Hierarchical clustering of enriched signaling pathways highlighted distinct expression patterns among DEGs across clusters. The proportions of each subpopulation were compared between normal and PD tissues.

### 2.17 Pseudotime trajectory analysis of cell subpopulations

Pseudotime trajectory analysis was performed on the subpopulations using Monocle (v2.30.1) (39) to investigate lineage dynamics. UMI count matrices were imported from Seurat objects to create CellDataSet objects using the “newCellDataSet” function. Statistical models were constructed via “estimateSizeFactors” and “estimateDispersions.” Dimensionality reduction was performed with DDRTree through the “reduceDimension” function, followed by trajectory ordering using “orderCells.” Branch-dependent gene expression modeling was also performed. The resulting trajectories delineated cell states, pseudotime, and potential lineage transitions.

### 2.18 Parkinson’s disease mouse modeling

Mouse modeling were performed following the standard methods (40, 41): Male C57BL/6J mice (9 weeks old) were randomly assigned to MPTP-treated (*n* = 8) and saline control (*n* = 8) groups. To model subacute Parkinson’s disease progression, mice received daily intraperitoneal injections of MPTP (30 mg/kg in saline) for 5 consecutive days. Control animals were injected with equivalent volumes of sterile saline. All mice were maintained in temperature-controlled dark chambers for 24 h post-injection to prevent hypothermia. Motor impairments were assessed on day 5 post-modeling. Midbrain substantia nigra tissues were harvested for: qRT-PCR, western blot and immunofluorescence.

### 2.19 RNA extraction and real-time PCR

Trizol reagent was used to extract total RNA following manufacturer instructions. RNA reversed transcription using PrimeScriptTM RT reagent Kit (YEASEN), and analyzed by quantitative PCR (qPCR) using SYBR Premix Ex TaqTM II (YEASEN) in ABI Q3 system. Relative gene expression was normalized to GAPDH. qPCR primers were as follows:

**Table 1:** 

Targets	Forward 5′ → 3′	Reverse 5′ → 3′
CA4	TACGTGGCCCCCTCTACTG	GCTGATTCTCCTTACAGGCTCC
Bmx	GCTCCCACTTTCCCAGAGAG	TTGGGGTAGAATGGCACCTG
Gapdh	AGGTCGGTGTGAACGGATTTG	GGGGTCGTTGATGGCAACA

### 2.20 Cell lysis solution and western blots

Tissue samples (100 mg wet weight) were homogenized in 1 mL ice-cold RIPA lysis buffer supplemented with 1 mM PMSF protease inhibitor. Protein concentrations were tested by BCA kit and equivalent proteins were loaded into SDS-PAGE. Following western blots were performed according to standard procedures. The primary antibodies were list as follow:

- Anti-CA4 (Proteintech, Cat#85706-1-RR).
- Anti-BMX (Proteintech, Cat#27413-1-AP).

### 2.21 Immunofluorescence

Paraffin-embedded tissue sections were subjected to standard deparaffinization and rehydration. Following antigen retrieval, sections were incubated with primary antibodies at 37 °C for 1 h under light-protected conditions. After three 5-min PBS washes, fluorescent secondary antibodies were applied at 37 °C for 30 min with light protection. Sections were then thoroughly rinsed with PBS buffer, counterstained with DAPI (5 min), and mounted for imaging. Fluorescence visualization was performed using a digital slide scanner (3DHISTECH, Hungary). The primary antibodies were list as follow:

- Anti-CA4 (Proteintech, Cat#85706-1-RR, 1:500).
- Anti-BMX (Proteintech, Cat#27413-1-AP, 1:200).

The secondary antibody were list as follow:

- Cy3-conjugated goat anti-rabbit IgG (H + L) (Beyotime, Cat#A0516).

### 2.22 Statistical analysis

All statistical analyses were performed using SPSS Statistics (Version 27.0; IBM Corp., Armonk, NY, United States). Data visualization was conducted with GraphPad Prism (Version 9.2; GraphPad Software, Inc., San Diego, CA, United States). Quantitative data underwent normality assessment via Shapiro–Wilk testing. Normally distributed variables are presented as mean ± standard deviation (SD) and compared between groups using two-tailed Student’s *t*-tests. Statistical significance was defined as *p* < 0.05.

## 3 Results

### 3.1 Identification of differentially expressed genes and enrichment analysis

**Figure 1:**
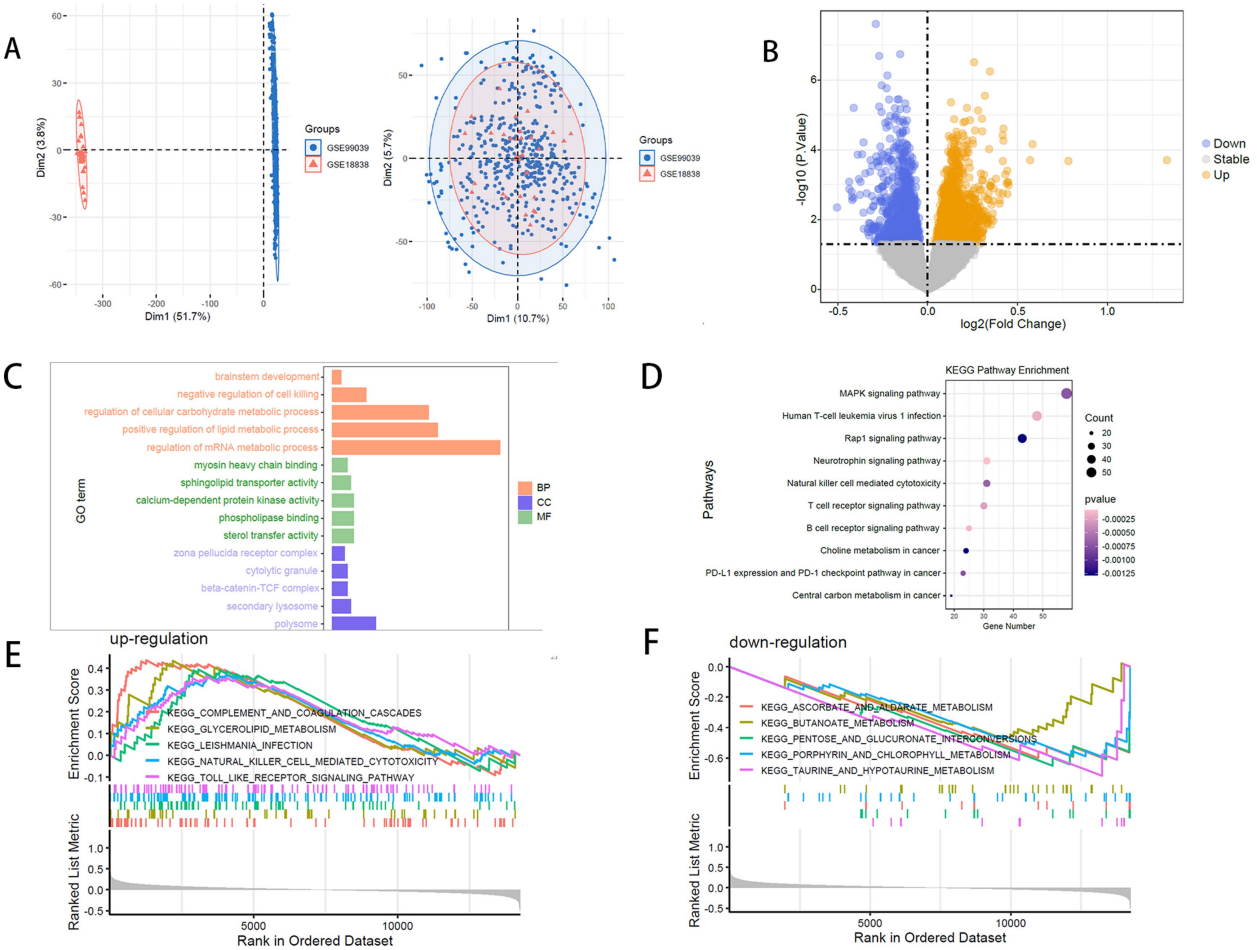
**(A)** sva batch effect removal: training set before and after batch correction. **(B)** Volcano plot of differentially expressed genes (blue: downregulated; yellow: upregulated). **(C)** GO enrichment analysis (bar plot). **(D)** KEGG enrichment analysis (bubble plot). **(E)** GSEA enrichment (upregulated gene sets). **(F)** GSEA enrichment (downregulated gene sets).

To mitigate batch effects between datasets GSE99039 and GSE18838, we performed batch correction and merged the two datasets, resulting in a combined cohort of 222 PD samples and 244 control samples (Figure 1A). Analysis of differential expression between PD and control groups revealed 2,221 DEGs, comprising 1,183 upregulated and 1,038 downregulated genes (Figure 1B).

GO and KEGG enrichment analyses were performed to investigate the biological functions and pathways linked to the DEGs displays the top five enriched terms for each GO category (Figures 1C,D). In the biological process category, DEGs showed significant enrichment in terms related to cellular carbohydrate metabolism regulation and brainstem development, indicating a strong link to metabolism and neural stem cell regulation. Significant enrichment was identified in the cytolytic granule, beta-catenin-TCF complex, and secondary lysosome within the CC category.

KEGG pathway analysis identified enrichment in immune response and metabolic pathways, such as choline metabolism in cancer, natural killer cell-mediated cytotoxicity, and central carbon metabolism in cancer. GSEA revealed that PD samples showed increased activity in immune-related pathways, including the Toll-like receptor signaling pathway and natural killer cell-mediated cytotoxicity, alongside decreased activity in metabolic pathways like ascorbate and aldarate metabolism (Figures 1E,F). These findings highlight the involvement of natural killer (NK) cell activity, immune dysregulation, and metabolic alterations in the pathogenesis of PD.

### 3.2 WGCNA identifies pathogenic genes interlinked with stem cell and metabolic genes

**Figure 2:**
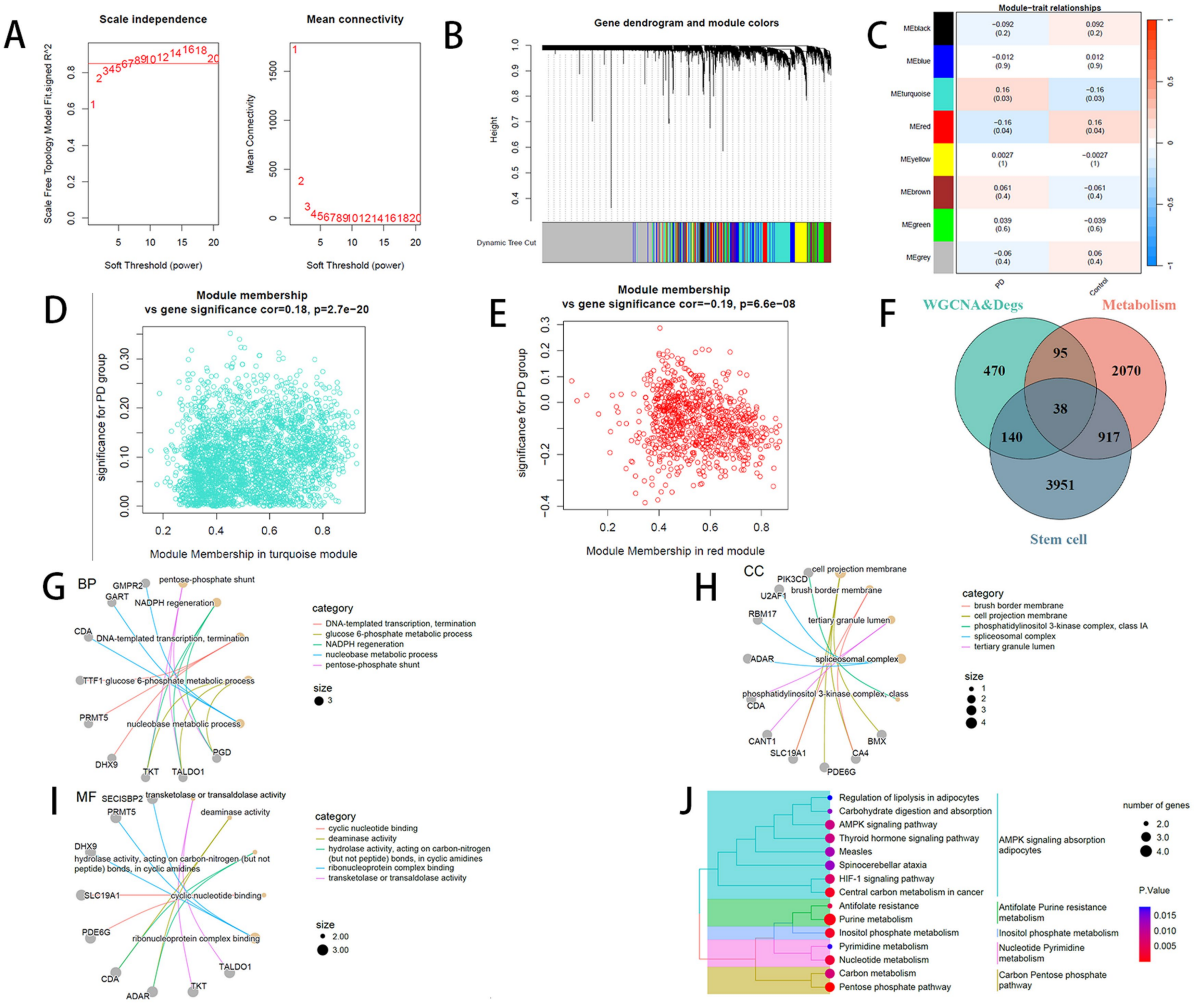
**(A)** Left: Selection of soft-thresholding power (*β*) for adjacency matrix. *X*-axis: soft-thresholding power; *Y*-axis: scale-free topology model fit (*R*^2^). Red line: cutoff threshold (*R*^2^ = 0.85). Right: Mean gene connectivity under different soft-thresholding powers. Red line: mean connectivity at selected *β*. **(B)** Hierarchical clustering dendrogram of co-expression modules (colors denote modules). **(C)** Module-trait associations heatmap. **(D)** Scatter plot of gene significance vs. module membership for the turquoise module. **(E)** Scatter plot of gene significance vs. module membership for the red module. **(F)** Overlap between WGCNA hub genes, DEGs, and stem cell/metabolism-related genes. **(G–J)** Enrichment analysis: **(G)** biological processes (BP); **(H)** cellular components (CC); **(I)** molecular functions (MF); **(J)** KEGG pathways.

WGCNA was conducted with PD and Control serving as phenotypic traits. The soft-thresholding power was determined to be 7, marking the initial point where the scale-free topology fit index (*R*^2^) achieved 0.85 (red line) (Figures 2A,B). Genes were clustered into modules using hierarchical clustering combined with dynamic tree cutting, resulting in seven modules excluding the grey module. Module-trait relationships were subsequently assessed, identifying the turquoise and red modules as most significantly correlated with phenotype (Figure 2C). Further correlation analysis between these two modules and phenotypic traits was performed (Figures 2D,E).

The 2,586 and 796 genes from the turquoise and red modules, respectively, were intersected with the upregulated and downregulated DEGs, yielding 609 and 134 overlapping genes. Merging these resulted in a total of 743 genes (Supplementary Figure S1). Based on the enrichment results highlighting stem cell and metabolic processes, we extracted 3,120 metabolism-related genes from the Molecular Signatures Database using the keyword “metabolism” and identified 5,046 stem cell-related genes from 26 gene sets in StemChecker. Intersection of these three gene sets identified 38 overlapping genes (Figure 2F).

Enrichment analyses of the 38 intersecting genes using the “clusterProfiler” package for GO and KEGG revealed a significant association with metabolic pathways (Figures 2G–J).

### 3.3 Development of a PPI network and application of machine learning for feature gene detection and ROC analysis

**Figure 3:**
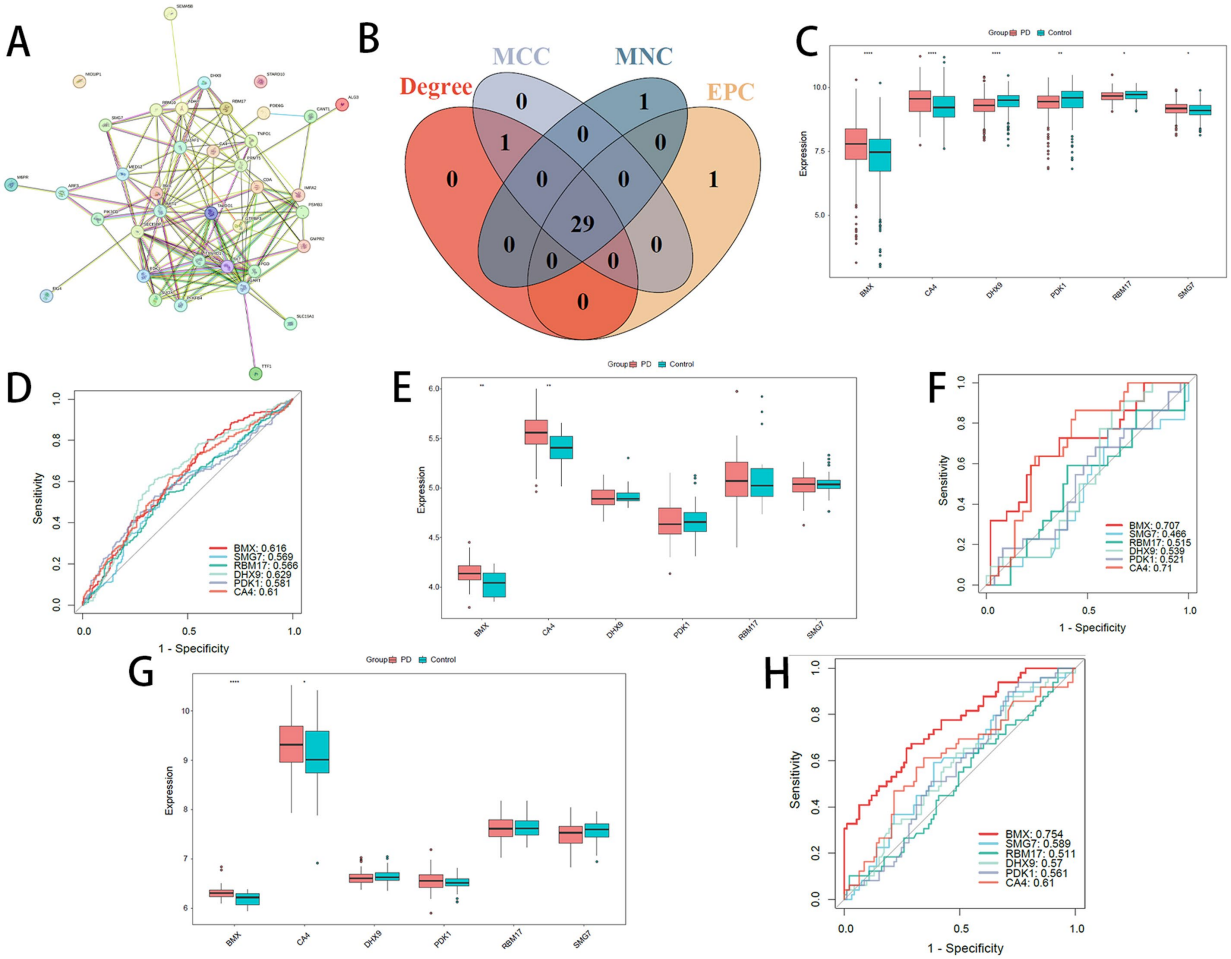
**(A)** PPI network of signature genes. **(B)** Venn diagram of top 30 hub genes identified by Degree/MCC/MNC/EPC algorithms. **(C)** Expression box plots of signature genes in training set. **(D)** ROC curves for signature genes in training set. **(E,G)** Expression box plots in validation sets: **(E)** GSE6613; **(G)** GSE57475. **(F,H)** ROC curves in validation sets: **(F)** GSE6613; **(H)** GSE57475.

PPI network for the specified genes was constructed using STRING, applying a minimum interaction score threshold of 0.15. Genes MID1IP1 and STARD10, which were absent from the network, were excluded, resulting in 36 genes retained for subsequent analyses (Figure 3A). The MCODE plugin was utilized for clustering analysis to detect densely connected regions (Supplementary Figure S2). Through the intersection of four distinct algorithms, 29 hub genes were identified (Figure 3B). Expression profiles of these shared model genes and PD status were extracted from both training and validation cohorts. Prognostic models were constructed by integrating 12 different machine learning algorithms in various combinations. Based on concordance indices (*C*-indices) across training and validation sets, a combined Stepwise GLM (both directions) and Random Forest (RF) model was selected for prognostic prediction (Supplementary Figure S3). This model achieved a *C*-index of 0.996 in the training set, 0.611 in GSE6613, and 0.902 in GSE57475, with an average *C*-index of 0.836. Ultimately, six feature genes were prioritized: DHX9, BMX, PDK1, CA4, SMG7, and RBM17. Notably, BMX and CA4 exhibited significant differential expression between PD and control groups in both training and validation cohorts (Figures 3C,E,G). Receiver operating characteristic (ROC) curve analyses further confirmed the strong predictive performance of these feature genes (Figures 3D,F,H). BMX and CA4 were therefore selected for downstream analyses.

### 3.4 Validation of BMX and CA4 upregulation in the substantia nigra of Parkinson’s disease model mice

**Figure 4:**
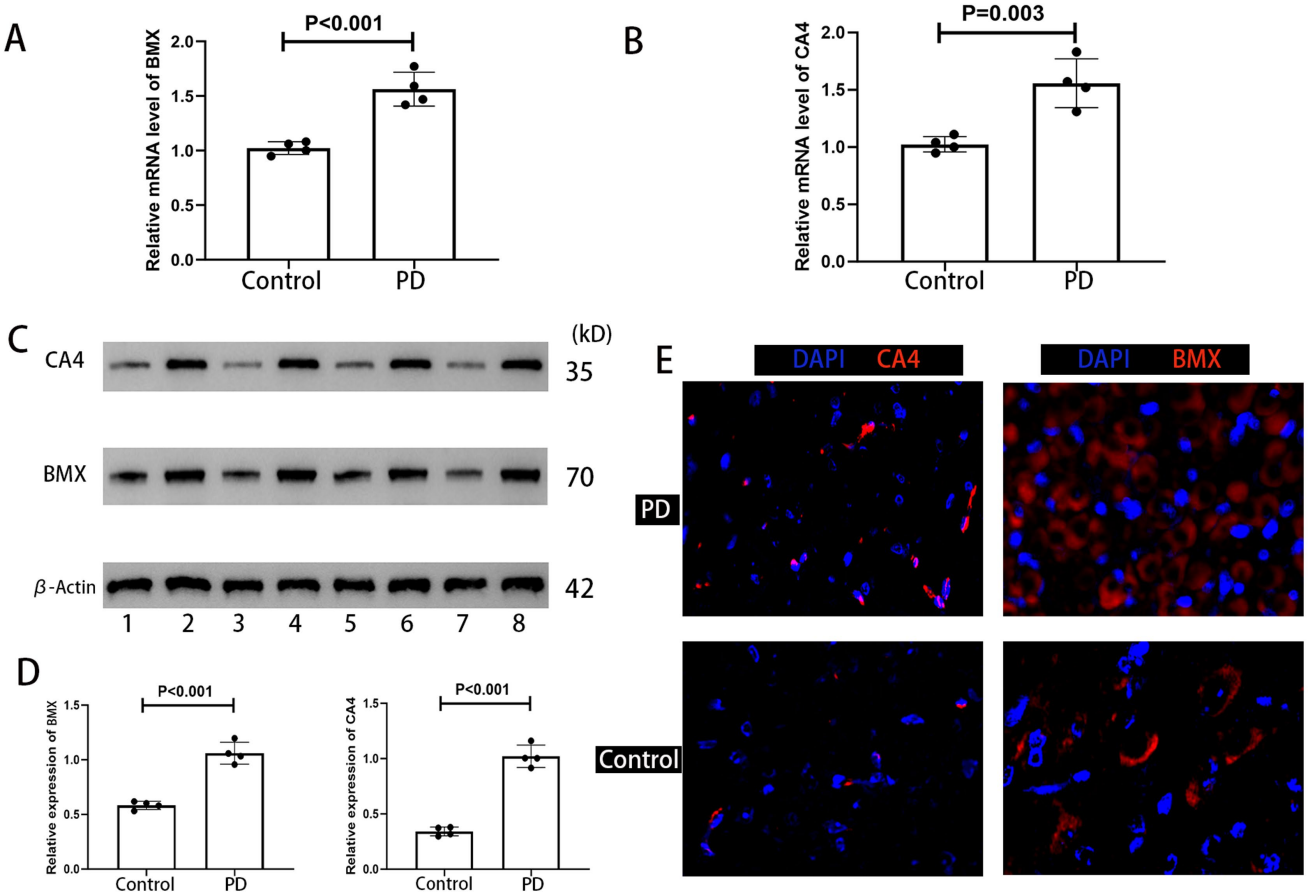
**(A,B)** qRT-PCR analysis of *BMX* and *CA4* mRNA expression (*n* = 4 mice/group). Data normalized to *GAPDH*. **(C,D)** Western blot quantification of CA4 and BMX protein expression. Loading control: β-actin. Representative blots shown above graphs [Biological replicates: Control group (Mice #1, 3, 5, 7), PD group (Mice #2, 4, 6, 8)]. **(E)** Immunofluorescence localization in substantia nigra sections. Nuclei counterstained with DAPI (blue). Target proteins: CA4 (red, left panels), BMX (red, right panels).

To verify bioinformatically predicted dysregulation of *BMX* and *CA4*, qRT-PCR analysis was performed on substantia nigra tissues from PD model mice and controls (*n* = 4 mice/group). Using *GAPDH* as endogenous control, relative mRNA expression was shown in Figures 4A,B: *BMX* (*p* < 0.001) and CA4 (*p* = 0.003) transcript levels increased in PD (*p* < 0.05). Western blotting further confirmed protein-level alterations,and quantification revealed significant upregulation in PD group (*p* < 0.001) (Figures 4C,D). Immunohistochemical analysis of tissue microarrays (*n* = 4/group) localized enhanced expression of both targets within nigral tissues of PD mice (Figure 4E).

### 3.5 Based on GSVA scoring and GSEA analysis of the two feature genes, followed by drug docking

**Figure 5:**
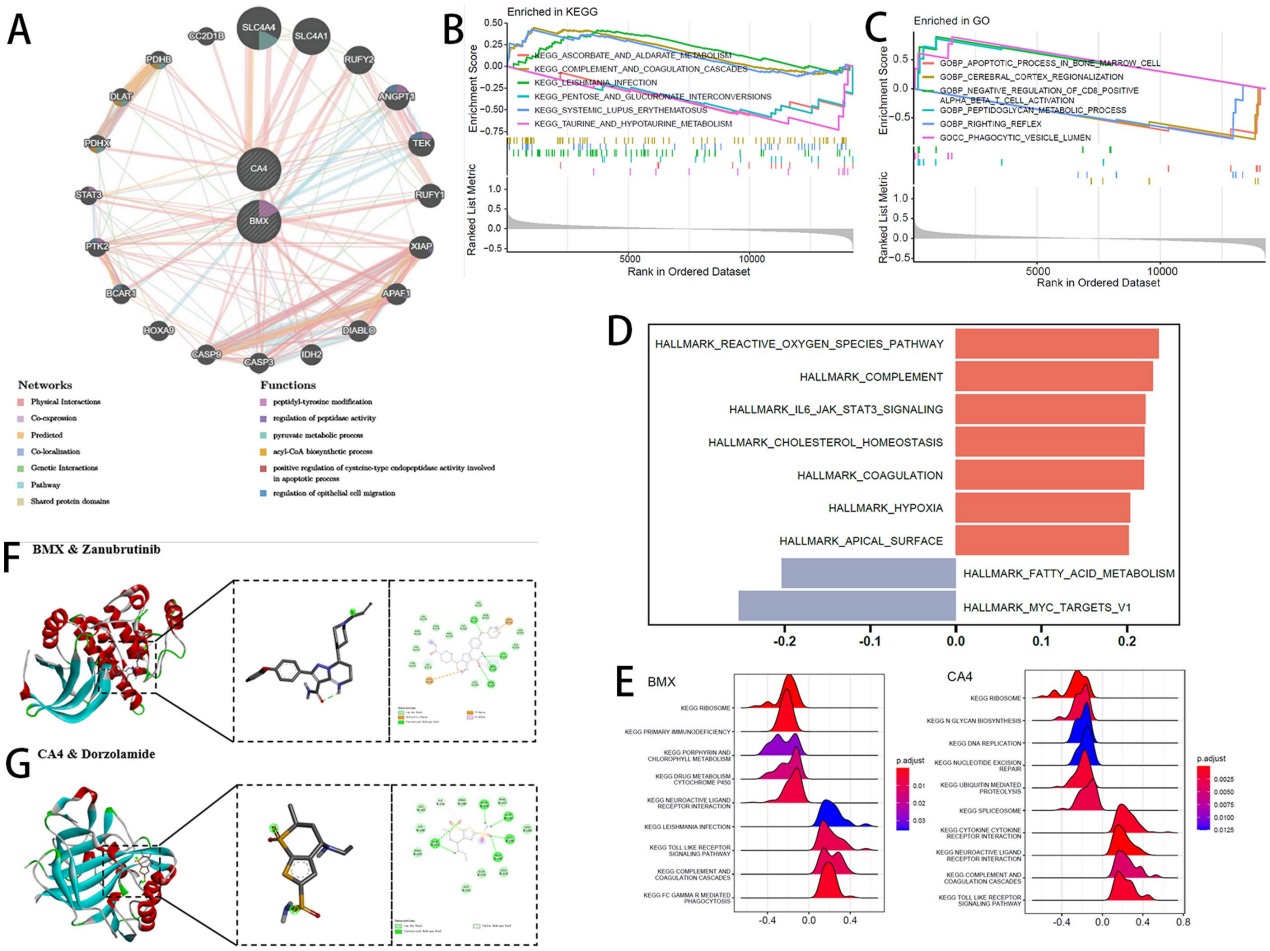
**(A)** GeneMANIA interaction network. **(B,C)** GSEA enrichment: **(B)** KEGG pathways; **(C)** GO terms. **(D)** GSVA correlation heatmap of enriched pathways. **(E)** Pathway enrichment for signature genes: **(A)** BMX; **(B)** CA4. **(F,G)** Molecular docking: **(F)** BMX with zanubrutinib; **(G)** CA4 with dorzolamide.

The GeneMANIA database was utilized to conduct a protein–protein interaction (PPI) analysis involving the two feature genes and 20 associated interacting genes (Figure 5A) predicting correlations among co-localization, shared protein domains, co-expression, and pathways. The genes were enriched in functions such as “peptidyl-tyrosine modification,” “regulation of peptidase activity,” and “pyruvate metabolic process.” Gene Set Enrichment Analysis (GSEA) of GO and KEGG pathways was performed to compare samples with high and low GSVA scores. A total of 22 KEGG pathways and 827 GO terms showed significant enrichment at a *p*-value threshold of 0.05. The top five upregulated and top five downregulated pathways are shown in Figures 5B,C. Using the GSVA algorithm, hallmark gene set enrichment scores were calculated for each sample, and correlations between the GSVA score and hallmark enrichment scores were assessed (Figure 5D).

Based on KEGG gene sets, GSEA revealed signaling pathways associated with the two feature genes under thresholds of adjusted *p* < 0.05 and |NES| >1 (Figure 5E). Both BMX and CA4 regulated the “RIBOSOME” pathway, but showed opposite regulatory trends in the “NEUROACTIVE LIGAND RECEPTOR INTERACTION” pathway.

For drug docking, compounds corresponding to the two feature genes were retrieved from DrugBank. The protein structures corresponding to BMX and CA4 were obtained from the PDB database (PDB IDs: 8X2A and 3F7B, respectively). BMX protein was docked with zanubrutinib (binding energy −6.24 kcal/mol) (Figure 5F), ritlecitinib (−5.06 kcal/mol), and fostamatinib (−5.05 kcal/mol) (Supplementary Figures S4A,B). CA4 protein was docked with topiramate (−4.86 kcal/mol), methazolamide (−4.63 kcal/mol) (Supplementary Figures S4C,D), and dorzolamide (−6.81 kcal/mol) (Figure 5G). Binding energies below −4.5 kcal/mol suggest spontaneous interactions with strong stability and affinity.

### 3.6 Model interpretation using SHAP and construction of diagnostic nomogram

**Figure 6:**
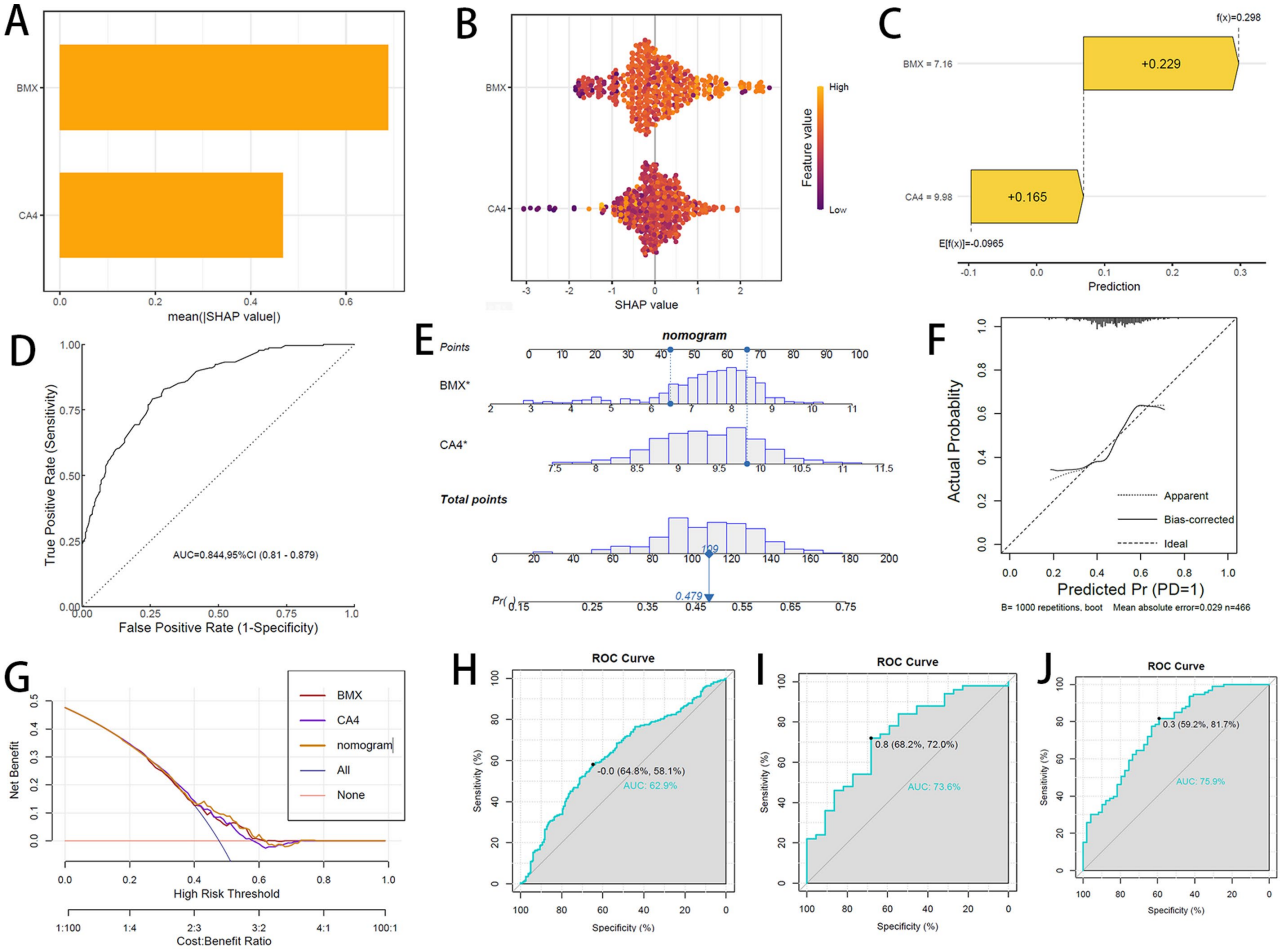
**(A)** SHAP global feature importance (bar plot). **(B)** SHAP multi-feature beeswarm plot. **(C)** SHAP force plot for individual prediction. **(D)** ROC curve of diagnostic model. **(E)** Diagnostic nomogram. **(F)** Calibration curve. **(G)** Decision curve analysis (DCA). **(H–J)** Nomogram ROC curves: **(H)** Training set; **(I)** GSE6613; **(J)** GSE57475.

The final predictive model was interpreted using the SHAP method, revealing that both BMX and CA4 contribute significantly to the global model variables (Figure 6A). The global distribution of SHAP values for both genes showed that higher expression levels predominantly correspond to positive SHAP values (right side of zero) (Figure 6B) suggests that elevated BMX and CA4 expression correlates with an increased risk of PD. The combined prediction using BMX and CA4 improved accuracy (Figure 6C), with ROC curves exceeding 0.8, demonstrating good predictive performance (Figure 6D).

To evaluate the combined diagnostic capability of BMX and CA4 for PD, both genes were incorporated into a nomogram (Figure 6E). Calibration and decision curve analyses confirmed the nomogram’s accuracy and clinical utility (Figures 6F,G). ROC curve analysis indicated that the nomogram attained AUC values exceeding 0.6 in both the training and validation cohorts (Figures 6H–J), indicating satisfactory predictive efficacy.

### 3.7 PD single-cell atlas and intercellular communication

**Figure 7:**
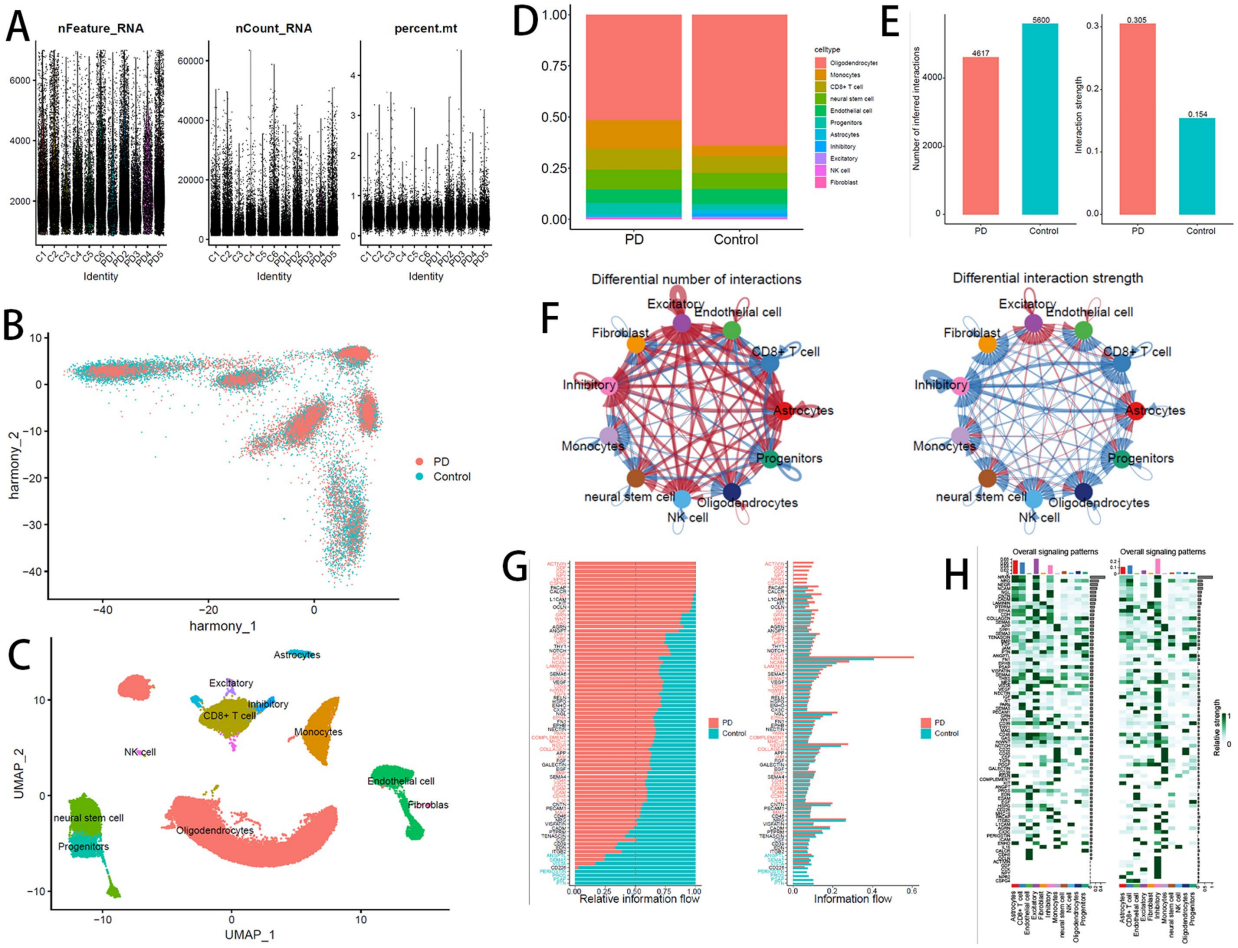
**(A)** Post-QC cell distribution. **(B)** Batch correction by Harmony. **(C)** Annotated UMAP cell atlas. **(D)** Cell type proportions. **(E)** Number and strength of cell–cell interactions mediated by signaling pathways (PD vs. Control). **(F)** Differential cell communication networks (red: PD; blue: control). **(G)** Pathway-specific communication strength (red: PD-enriched; blue: control-enriched; black: neutral). **(H)** Signaling intensity heatmap (left: PD; right: control). Color depth indicates interaction strength. Top/right bars: cumulative signaling intensity.

Based on the single-cell dataset GSE157783, comprising 5 PD samples and 6 Control samples, quality control was performed (Figure 7A), yielding 41,189 high-quality cells for subsequent analysis. PCA was performed on the 2,000 most variable genes, and batch effects were corrected using Harmony (Figure 7B). The UMAP algorithm was utilized for dimensionality reduction and clustering, resulting in 20 unique cell clusters. These clusters were annotated into 10 cell types, with their respective proportions shown in Figure 7C: oligodendrocytes, monocytes, CD8^+^ T cells, neural stem cells, endothelial cells, progenitors, astrocytes, inhibitory neurons, excitatory neurons, NK cells, and fibroblasts (Figure 7D).

Cell–cell communication analysis was conducted across all cell populations (Figures 7E,F), alongside evaluation of signaling pathways and ligand–receptor pair activation involved in cellular interactions (Figure 7G), as well as differences in activation between PD and Control groups. Intercellular communication was predicted based on specific pathways and ligand–receptor pairs (Figure 7H). The results revealed that although the number of communicating cells decreased in the PD group, the communication intensity was markedly increased, particularly among excitatory neurons, astrocytes, CD8^+^ T cells, and NK cells. Inflammation-related pathways were notably activated in PD samples.

### 3.8 Expression patterns of feature genes and corresponding changes in cell subclusters in single-cell data

**Figure 8:**
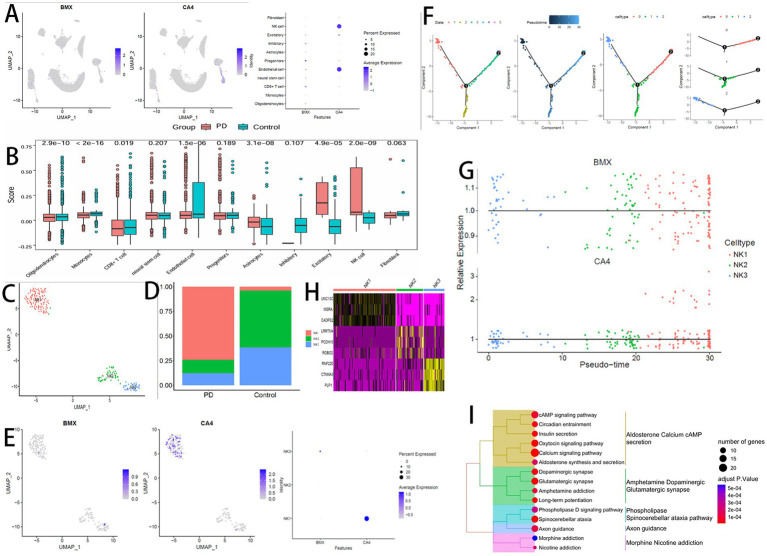
**(A)** Expression of signature genes in single cells (dot plot). **(B)** ssGSEA scores based on 6 signature genes. **(C)** NK cell subclusters (UMAP). **(D)** Differential abundance of NK subclusters (PD vs. Control). **(E)** Signature gene expression in NK cells (dot plot). **(F)** Pseudotime trajectory of NK cells. **(G)** Dynamic expression of signature genes along pseudotime. **(H)** Marker gene heatmap for NK subclusters. **(I)** KEGG enrichment of NK1 cluster-specific hallmarker genes.

We analyzed the expression patterns of BMX and CA4 genes in the single-cell dataset (Figure 8A). BMX was found to be broadly expressed across all 10 cell types, whereas CA4 showed high expression specifically in NK cells and endothelial cells. We calculated the ssGSEA scores of six feature genes (DHX9, BMX, PDK1, CA4, SMG7, and RBM17) in the annotated cell populations (Figure 8B), indicating that NK cells showed the most pronounced differential expression compared to other cell types. Therefore, NK cells were selected as the core cell population for subsequent analyses.

Re-clustering of NK cells revealed three distinct subclusters, designated as NK1, NK2, and NK3 (Figure 8C). Comparing the proportions of these subclusters between PD and Control groups revealed that NK2 and NK3 proportions were significantly lower in PD, whereas NK1 was significantly enriched in PD samples (Figure 8D). Expression analysis within these subclusters showed that CA4 was highly expressed in the NK1 subcluster (Figure 8E).

We further extracted NK subpopulations and performed pseudotime trajectory analysis using Monocle2 (Figure 8F). Tracking the expression dynamics of feature genes along the pseudotime trajectory indicated a differentiation trajectory oriented toward the NK1 subcluster. Scatter plots of gene expression levels confirmed notably higher CA4 expression in NK1 compared to NK2 and NK3 (Figure 8G).

To clarify the functional characteristics of NK subclusters, we combined gene heatmap data (Figure 8H) and observed that NK1 cells highly express genes such as UNC13C, CADPS2, and MSRA, which are involved in processes like neuron- and endocrine cell-mediated secretory granule exocytosis, antioxidant maintenance of mitochondrial function, and cell survival. KEGG enrichment analysis of the hallmark genes specifically expressed in NK1 (Supplementary Figure S5) demonstrated enrichment in pathways including calcium-cAMP secretion, dopaminergic and glutamatergic synapse, and axon guidance (Figure 8I), highlighting the role of NK1 cells in cytotoxic granule release, synapse formation, and axon guidance.

## 4 Discussion

PD involves complex pathological mechanisms, including dopaminergic neuron degeneration, α-synuclein aggregation, neuroinflammation, mitochondrial dysfunction, and various interacting processes (42). This study investigates PD through metabolic and stem cell perspectives, employing scRNA-seq and extensive bulk RNA-seq data for comprehensive bioinformatics analysis. Our study highlights the pivotal role of the CA4 gene and NK cells in PD development and progression, offering novel insights into its pathogenesis and potential therapeutic targets. The following discussion focuses on our core findings, innovations, and translational potential.

Initially, RNA-seq data was utilized to identify DEGs between PD and control groups. Subsequent GO functional annotation, KEGG pathway enrichment, and GSEA analyses suggested involvement in brainstem cell development and metabolic processes. By intersecting the DEGs with PD-related up- and down-regulated gene modules identified through WGCNA, we identified 743 genes. Further intersecting with stem cell- and metabolism-related genes yielded 38 genes. GO and KEGG enrichment of these 38 genes again emphasized metabolism-related pathways, with no significant enrichment in stem cell-associated pathways, suggesting that endogenous neural stem cells may not play a central role in PD progression.

Utilizing PPI network construction and integrated machine learning techniques, we refined the initial set of 38 genes to six key genes: DHX9, BMX, PDK1, CA4, SMG7, and RBM17. Among these, only BMX and CA4 showed significant differential expression in both training and validation datasets. Our characteristic genes demonstrated good performance in ROC curve analysis, SHAP values, and nomogram models. The combined diagnostic model based on these two genes showed high accuracy and predictive power. Given that clinical diagnosis of PD remains challenging in early stages due to lack of reliable biomarkers despite clear clinical manifestations (43), our findings contribute to building a more precise and comprehensive diagnostic model for PD. Additionally, we identified potential drug targets and corresponding compounds for these two genes through the DrugBank database, laying a foundation for subsequent drug intervention studies.

In the scRNA-seq atlas, we identified 10 core cell types and performed cell–cell communication analysis. Although the number of communicating cells decreased in the PD group, the communication strength was significantly enhanced, with more frequent interactions among excitatory neurons, astrocytes, CD8^+^ T cells, and NK cells. Inflammation-related pathways showed significant activation in PD. The activation of glial cells and inflammatory cells, as well as their interactions with neurons, are crucial in the onset and progression of PD (44–46), which aligns with current research findings.

In our scRNA-seq analysis of BMX and CA4 gene expression patterns, we found that BMX was broadly expressed across all 10 cell types, while CA4 showed high expression in NK cells and endothelial cells. The ssGSEA scores from six characteristic genes indicate that NK cells are crucial in PD pathogenesis.

Traditionally, neuroinflammation in PD has been mainly attributed to microglia and T cells (47–49), while the role of NK cells has long been overlooked. Our findings challenge this paradigm. Recent single-cell sequencing studies have further revealed significant phenotypic and functional alterations of NK cell subsets in the peripheral blood and cerebrospinal fluid of PD patients, indicating their potential association with disease progression (50, 51). Previous studies reporting increased NK cell numbers mostly sampled peripheral blood and cerebrospinal fluid, whereas our study used midbrain substantia nigra tissue for single-cell sequencing, providing a more precise conclusion. However, the specific mechanisms and regulatory networks of NK cells in PD remain controversial and require further in-depth investigation.

We performed reclustering of NK cells and identified three subclusters. Pseudotime analysis of NK cells revealed a differentiation trajectory toward the NK1 subset. In the UMAP plot, the CA4 gene was highly expressed in NK1 cells. This result suggests a key role for CA4 in the progression of PD. Previous studies have indicated that NK cells may have a double-edged sword effect in PD progression: they can contribute to pathological protein clearance through immune surveillance, thereby inhibiting disease development, but excessive activation may lead to direct damage of vulnerable dopaminergic neurons via the release of granzyme B (52, 53).

In this study, gene heatmap results and KEGG pathway enrichment analysis of differential genes in NK1 cells indicated involvement in secretory granule exocytosis, cAMP signaling, calcium signaling, dopaminergic neuron synapse formation, and axon growth. These findings suggest that NK1 cells may participate simultaneously in cytotoxic activity and neuroprotection. CA4 (carbonic anhydrase IV), as a membrane protein, typically acts to alleviate acidic environments or maintain pH homeostasis when its activity or expression is increased (54), rather than directly causing acidification. We hypothesize that CA4 upregulation is crucial for NK cell survival in the acidic microenvironment of PD, sustaining their physiological functions and enhancing migration.

Analysis revealed a significant increase in resting NK cells in the PD group compared to controls, with a negative correlation between CA4 expression and resting NK cells (Supplementary Figure S6). This suggests that increased CA4 expression may help hinder PD progression. Previous studies on midbrain dopaminergic neuron lineages have shown that dopaminergic progenitor cells can differentiate into dopaminergic neurons and glutamatergic neurons (55). Our findings indicate that NK1 cells also influence the formation of both glutamatergic and dopaminergic neurons, implying that NK1 cells may affect the differentiation outcomes following exogenous stem cell transplantation.

This study has certain limitations: (1) the scRNA-seq data sample size is relatively small. (2) The regulatory mechanisms of characteristic genes in NK cells are not yet fully understood, current experimental evidence cannot demonstrate PD-specificity of this CA4-NK1-PD axis, nor can it preclude its potential critical role in other neurodegenerative conditions such as Alzheimer’s disease, highlighting a crucial area for future research.

## 5 Conclusion

Collectively, our study demonstrates that CA4 plays a pivotal role in Parkinson’s disease pathogenesis. We further identified and characterized the disease-associated NK1 cellular subset, unveiling previously unrecognized neuroimmune mechanisms. These findings enabled the development of a high-accuracy diagnostic model and therapeutic compound prediction platform, revealing CA4-NK1-PD axis as a promising target for future interventions.

## Data Availability

The original contributions presented in the study are included in the article/Supplementary material, further inquiries can be directed to the corresponding author.

## Footnotes

^1^ https://go.drugbank.com
