# Supplementary material for: Metabolic-stem cell crosstalk in PD: NK1 cells as key mediators from a bioinformatics perspective
Source: Front Neurol. 2025 Nov 13;16:1681261. doi: 10.3389/fneur.2025.1681261 (PMC12657439; doi:10.3389/fneur.2025.1681261)
Supplement: Supplementary file 1 [file Data_Sheet_1.pdf]

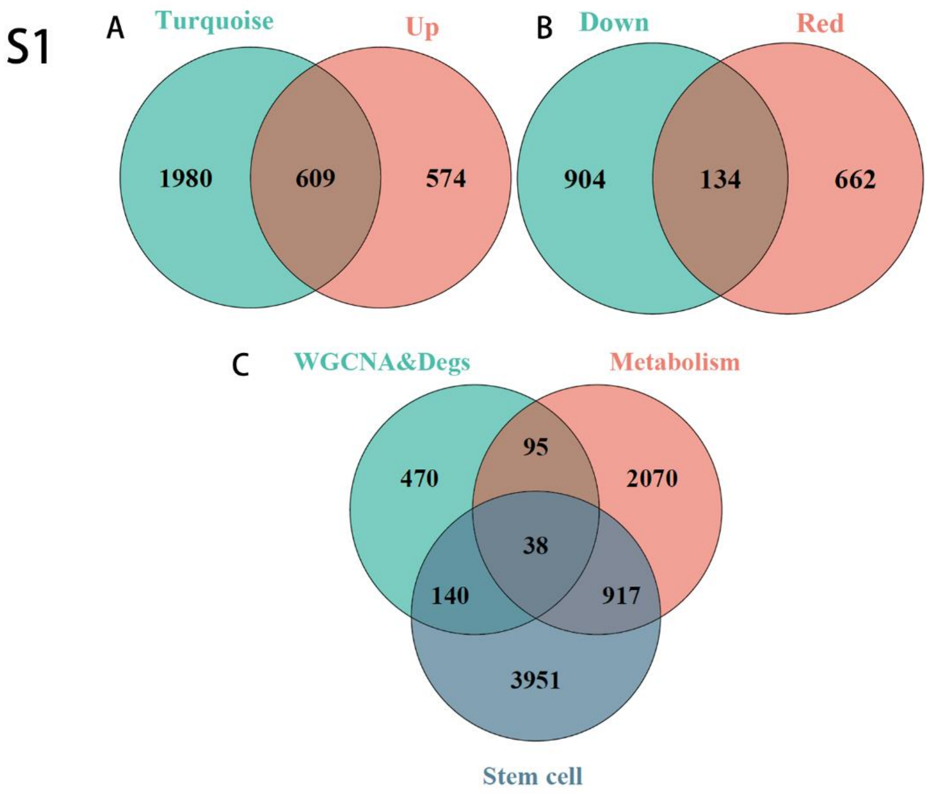

Supplementary 1

(A) Venn diagram showing intersection between PD-upregulated genes and turquoise module genes from WGCNA.

(B) Overlap of PD-downregulated genes with the red module genes identified by WGCNA.

(C) Four-way intersection analysis: Genes common to WGCNA modules, differentially expressed genes (DEGs), and functionally annotated stem cell/metabolism-related genes.

S2

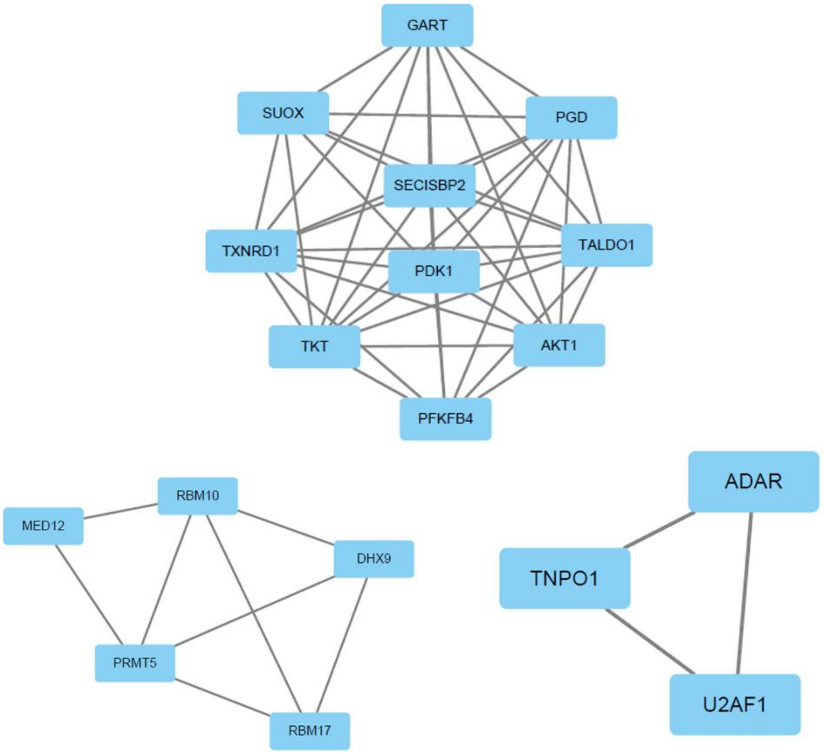

Supplementary 2. **Functional modules in protein-protein interaction (PPI) network identified by MCODE**

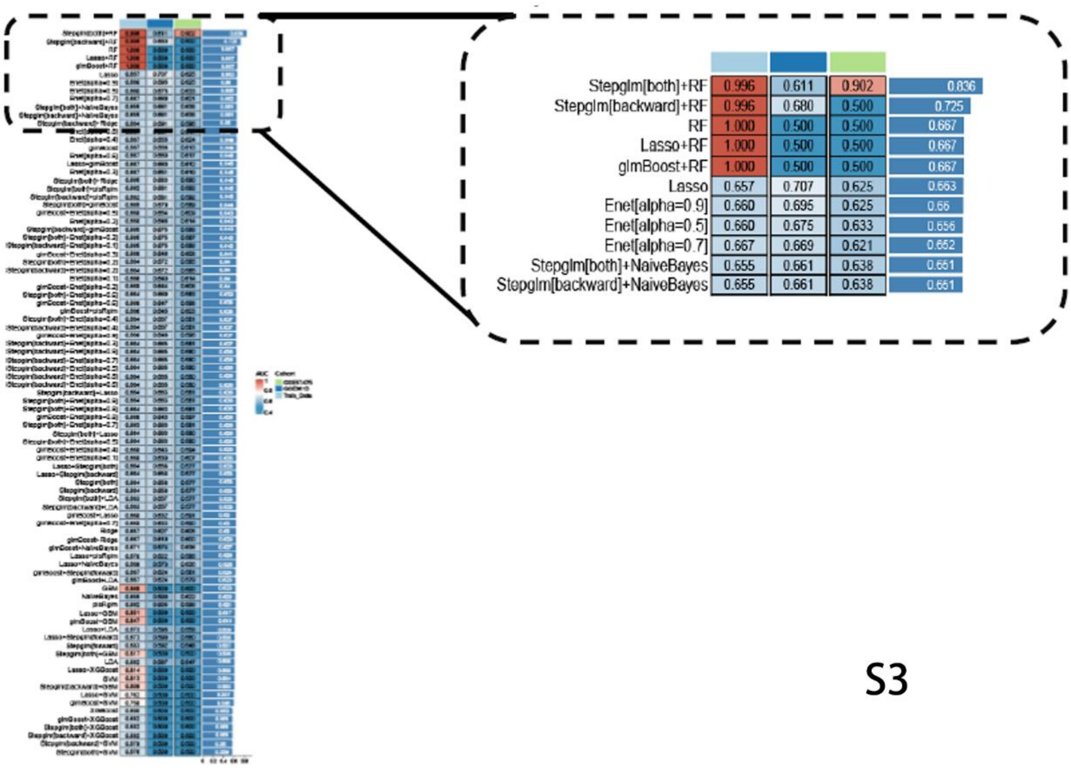

Supplementary 3. **Comparative analysis of 12 integrated machine learning algorithms**

(Training set performance and Validation set performance. Heatmaps quantify C-indices)

S4

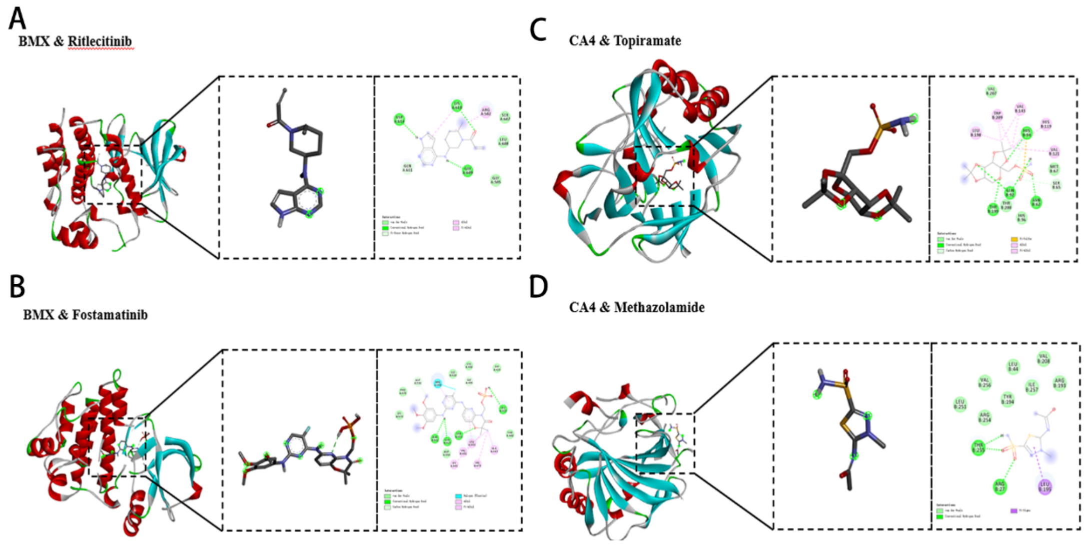

Supplementary 4. **Molecular docking analysis of kinase inhibitors**

(A) BMX-Ritlecitinib binding pose. (B) BMX-Fostamatinib interaction. (C) CA4-Topiramate complex. (D) CA4-Methazolamide binding site.

S5

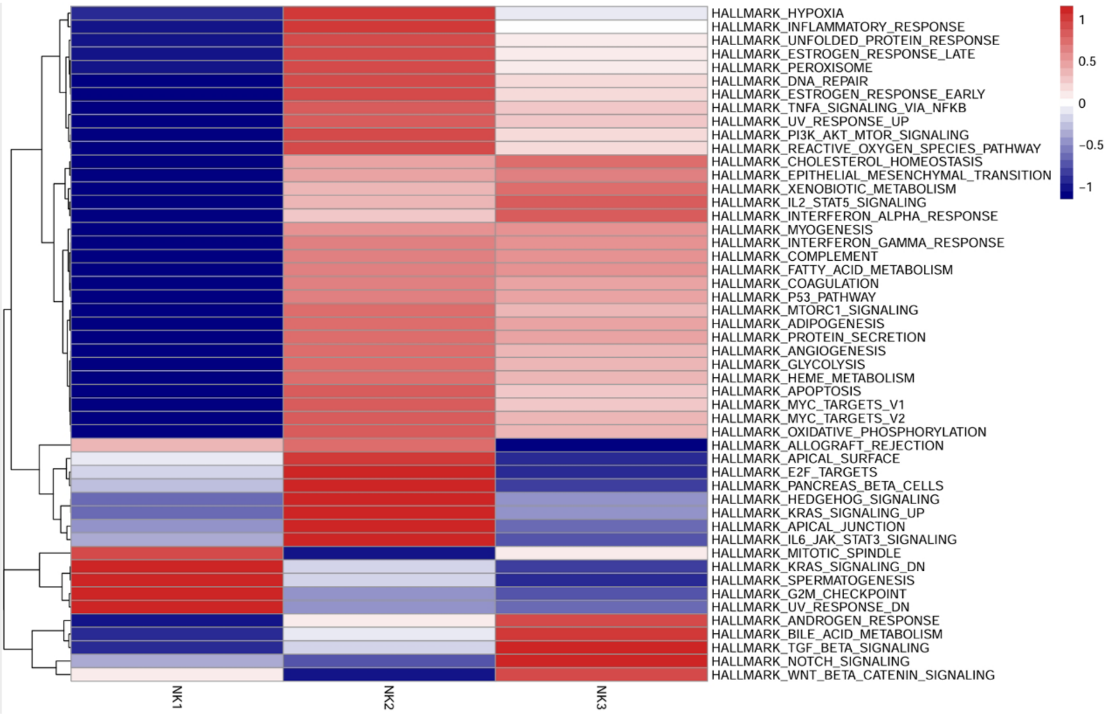

Supplementary 5. **Hallmark pathway signatures in NK subclusters**

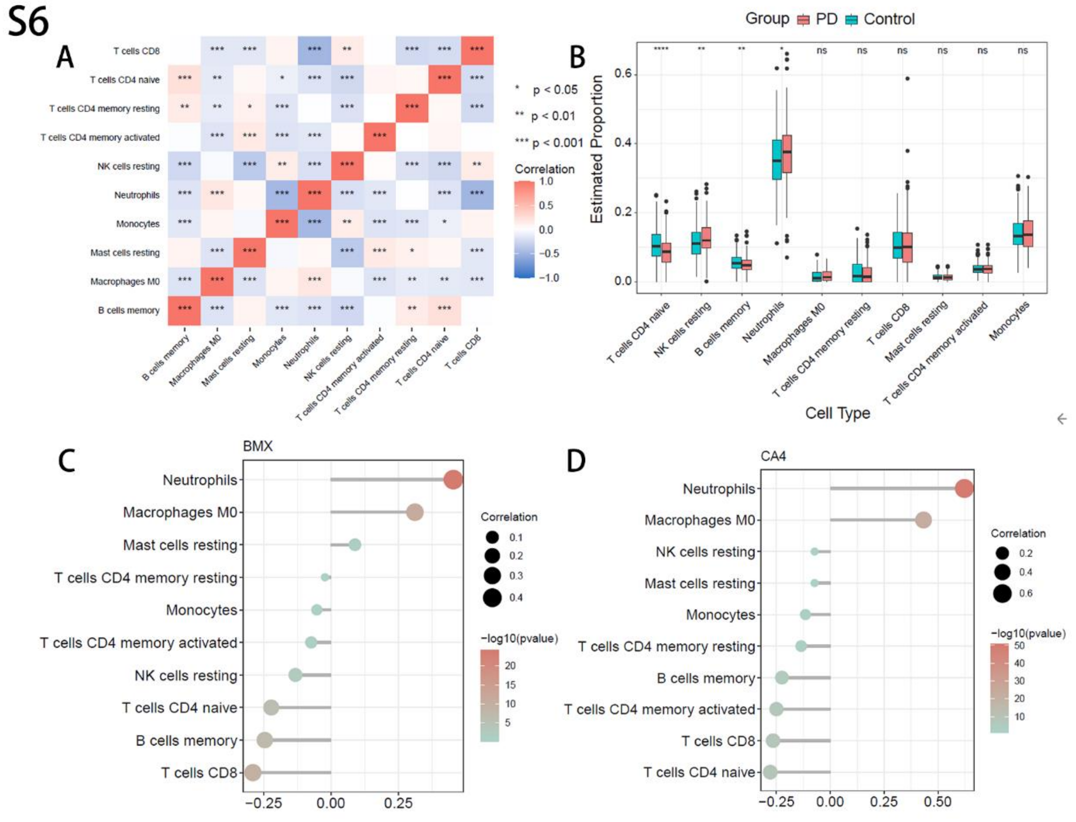

Supplementary 6. **Immune cell profiling in PD pathogenesis**

(A) Intercellular correlation heatmap. (B) Immune cell differences (PD vs Ctrl). (C-D) BMX/CA4 immunomodulatory correlations.
